# Supplementary material for: Vagus Nerve Stimulation Modulates Inflammation in Treatment-Resistant Depression Patients: A Pilot Study
Source: Int J Mol Sci. 2024 Feb 26;25(5):2679. doi: 10.3390/ijms25052679 (PMC10931975; doi:10.3390/ijms25052679)
Supplement: Supplementary file 1 [file ijms-25-02679-s001.zip › ijms-2825308-supplementary.pdf]

ID	Date of surgery	years from diagnosis	Supplements	Diet	Smoking	BMI	Sample collection date	VNS Parameters
1	19/11/2007	11.00	Centrum, Metamucil, Catvate	Regular	No	27.77	18/08/2015	2.0 mA, 30 Hz, 500 µs, 30 s/5 min
2	03/06/2008	2.00	None	Regular	11-25 cigarettes/day	25-30	09/06/2015	1.25 mA, 30 Hz, 250 µs, 30 s/5 min
3	13/08/2008	2.00	Oméga 3	Regular	No	25-30	01/09/2015	1.0 mA, 30 Hz, 500 µs, 30 s/5 min
4	20/10/2008	5.00	None	Regular	< 25 cigarettes /day	25-30	17/08/2015	2.0 mA, 30 Hz, 250 µs, 30 s/5 min
5	30/10/2009	1.00	Omega 3	Regular	No	21.11	18/08/2015	0.5 mA, 20 Hz, 250 µs, 30 s/5 min
6	06/04/2010	15.00	None	Regular	No	30.11	14/04/2015	1.75 mA, 30 Hz, 250 µs, 30 s/5 min
7	24/11/2010	6.00	None	Regular	25 cigarettes/day	30-35	21/07/2015	2.25 mA, 25 Hz, 250 µs, 30 s/3 min
8	03/11/2014	20.00	None	Regular	No	37.66	04/05/2015	1.25 mA, 30 Hz, 500 µs, 30 s/5 min

Table S1: Clinical, biological characteristics and VNS settings of the patients
